# The Proliferation–Invasion Dichotomy: Transcriptomic Signatures of lncRNA and Ubiquitin–Proteasome Networks in Hypermutated Endometrial Cancer

**DOI:** 10.3390/ijms27208954

**Published:** 2026-10-09

**Authors:** João Casanova, Ana Gomes da Costa, Marta Tripepi, Ana Pestana, Ana Catarino, Nélson Martins, Luís Teixeira, Nadeem Abu-Rustum, Rita Rosado-Ramos, Rune Matthiensen, Jorge Lima

**Affiliations:** 1 Gynecologic Oncology Unit, Department of Obstetrics and Gynecology, Hospital da Luz Lisboa, Luz Saúde, Avenida Lusíada 100, 1500-650 Lisboa, Portugal; joaomiguelcasanova@gmail.com (J.C.); ana.g.costa@hospitaldaluz.pt (A.G.d.C.); 2 Unit of Gynecology and Obstetrics, Department of Women and Children, University of Padua, 35122 Padua, Italy; marta.tripepi@studenti.unipd.it; 3 Department of Pathology, Hospital da Luz Lisboa, Luz Saúde, Avenida Lusíada 100, 1500-650 Lisboa, Portugal; ana.pestana.lopes@hospitaldaluz.pt (A.P.); acatarino@hospitaldaluz.pt (A.C.); 4 Católica Biomedical Research Centre, Faculdade de Medicina, Universidade Católica Portuguesa, 2780-156 Oeiras, Portugal; nemartins@ucp.pt (N.M.); luteixeira@ucp.pt (L.T.); 5 Gynecology Service, Department of Surgery, Memorial Sloan-Kettering Cancer Center, Weill Medical College of Cornell University, 1275 York Avenue, New York, NY 10065, USA; abu-rusn@mskcc.org; 6 Hospital da Luz Learning Health, Luz Saúde, Avenida Lusíada 100, 1500-650 Lisboa, Portugal; rita.joao.ramos@luzsaude.pt; 7 iNOVA4Health, NOVA Medical School, Faculdade de Ciências Médicas, Universidade NOVA de Lisboa, 1169-056 Lisboa, Portugal; rune.matthiesen@nms.unl.pt; 8 Department of Obstetrics and Gynecology, Hospital da Luz Lisboa, Luz Saúde, Avenida Lusíada 100, 1500-650 Lisboa, Portugal; 9 Comprehensive Health Research Centre (CHRC), NOVA Medical School (NMS), Universidade NOVA de Lisboa, Campo Mártires da Pátria, 130, 1169-056 Lisboa, Portugal

**Keywords:** endometrial cancer, ubiquitin–proteasome system (UPS), microsatellite instability (MSI), POLE hypermutation, proteostasis, deubiquitinase (DUB)

## Abstract

Endometrial cancer incidence is markedly rising globally, posing an urgent need to elucidate the molecular mechanisms driving disease characterization and progression. Endometrial cancer evolution is driven by canonical Wnt and p53 pathway alterations, yet the mechanisms orchestrating the transition from localized proliferation to stromal invasion, the proliferation–invasion dichotomy, remain poorly understood. While long non-coding RNAs (lncRNAs) are emerging as critical correlates of divergent tumor phenotypes, their coordination with the broader ubiquitin–proteasome system (UPS), specifically E3 ligase and deubiquitinase (DUB) networks, during endometrial cancer progression is largely unexplored. Whole-transcriptome RNA sequencing was performed on formalin-fixed paraffin-embedded (FFPE) tissue from a clinical cohort of 38 patients, comprising 34 primary endometrial carcinomas across distinct molecular subtypes (MSI, POLE, p53abn, and NSMP) and four pre-cancerous endometrial intraepithelial neoplasia (EIN) lesions. To stringently control for baseline patient-specific confounding, differential gene expression and principal component analyses were executed using a strict, intra-patient, matched-pair design (Tumor vs. Adjacent Normal). A principal component analysis of the batch-corrected transcriptome revealed a profound divergence between tumor and paired adjacent normal phenotypes. This spatial divergence was heavily characterized by lncRNA signatures (including *SOCS2-AS1*) strongly associated with extracellular matrix (ECM) remodeling. Concurrently, we identified a critical, malignancy-specific transcriptomic dysregulation across the broader UPS network. Within this altered transcriptional landscape, the ubiquitin C-terminal hydrolase (UCHL) family emerged as a highly significant node. *UCHL5* displayed robust, global upregulation across the pan-cancer cohort compared with matched controls (log_2_FC = 0.83, *p*adj < 0.001). Strikingly, *UCHL3* exhibited global upregulation (log_2_FC = 1.23, *p*adj = 0.002) that was highly concentrated within the microsatellite instability (MSI) and polymerase epsilon (POLE) hypermutated subtypes, highlighting a potential compensatory transcriptomic response to the predicted cellular stress inherent to high mutational burdens. In an exploratory subset of pre-cancerous EIN lesions (n = 4), neither the invasive lncRNA networks nor profound UPS/UCHL dysregulation were observed, suggesting a preliminary trend that requires validation in larger precursor cohorts. Our paired transcriptomic mapping reveals that endometrial cancer progression is characterized by divergent transcriptomic programs: specific lncRNA networks are tightly coupled to the invasive remodeling of the ECM, while concurrent dysregulation of broad UPS networks reflects the altered proteostasis associated with this phenotypic shift. The pronounced upregulation of critical UPS nodes, such as UCHL3 in MSI/POLE tumors, highlights a landscape of novel, enzymatically targetable vulnerabilities in hypermutated endometrial cancers.

## 1. Introduction

The incidence and associated mortality of endometrial cancer (EC) are rising [1]. Worldwide, EC is the sixth most common cancer in females, with roughly 417,000 new cases and 97,000 attributable deaths in 2020 [2]. The two major factors contributing to the increasing incidence of EC are population aging and the increased prevalence of obesity [3,4]. Classically, EC was categorized into two types: Type I, or endometrioid (accounting for approximately 80% of all cases), and Type II, or non-endometrioid (accounting for roughly 20% of all cases) [1,5]. Endometrioid EC develops due to excess unopposed estrogen and often emerges from a background of atypical endometrial hyperplasia. In contrast, non-endometrioid ECs comprise several histologic subtypes, including serous, clear-cell, carcinosarcoma, transitional cell carcinoma, squamous-cell carcinoma, and undifferentiated carcinoma [6]. These tumors are generally hormone-independent, and most lack well-defined precursor lesions. However, recent evidence indicates that serous endometrial intraepithelial carcinoma represents an aggressive precursor to endometrial serous carcinoma [7]. In addition to this categorization, EC is usually classified using a three-tier system developed by the International Federation of Obstetrics and Gynecology (FIGO) [8]. This system grades tumors from one to three, based on the proportion of glandular versus solid components within the tumor [8]. Grade 1 (G1) and Grade 2 (G2) tumors are termed low-grade and are generally associated with a more favorable prognosis [8]. In contrast, Grade 3 (G3) tumors are considered high-grade malignancies and exhibit heterogeneous clinical behavior [9,10]. Following the landmark trial published by The Cancer Genome Atlas (TCGA) Consortium, molecular classification is currently replacing classical classification systems [11]. The seminal TCGA trial revolutionized research in EC by classifying the disease into four distinct molecular subtypes: polymerase epsilon ultramutated (POLE), microsatellite instability hypermutated (MSI), copy-number low, and copy-number high [11]. Additionally, this study showed that oncologic outcomes, specifically progression-free survival, differed across molecular categories [11]. Since this trial, research groups have developed more cost-effective strategies to characterize the four molecular groups in the clinical setting [12,13,14]. The POLE group requires next-generation sequencing to determine mutational status; however, the MSI subgroup (involving deficiency in one or more mismatch repair proteins [MMRd] and the copy-number high group (characterized by p53 mutations) are currently classified using immunohistochemical surrogates [15,16]. Finally, the copy-number-low subgroup, also designated as no specific molecular profile (NSMP), lacks specific surrogates and is diagnosed by exclusion (*POLE* and *TP53* wild type and no MMRd) [17]. The molecular profiling of EC carries an increasing clinical impact, and a recently published trial demonstrated its implications for guiding adjuvant therapy [18]. As aforementioned, the landmark TCGA trial opened several fields of research. Subsequently, in 2020, an extensive multiomic analysis of a cohort of EC specimens was published [19]. The authors identified additional consequences of perturbations in the p53 and Wnt/β-catenin pathways, suggested a role for circular RNAs in the epithelial–mesenchymal transition, and provided further insight into proteomic markers associated with clinical and genomic tumor subgroups [19]. Overall, this integrative multiomic analysis highlighted novel molecular associations relevant to endometrial tumorigenesis and uncovered potential therapeutic targets [19]. Post-translational modifications of proteins play a key role in a wide variety of cellular functions. Ubiquitination, which is an essential protein post-translational modification, takes place through the sequential action of ubiquitin-activating enzymes (E1s), ubiquitin-conjugating enzymes (E2s), and ubiquitin ligase enzymes (E3s) [20]. In contrast to these enzymes, deubiquitinating enzymes (DUBs) remove ubiquitin from target proteins and disassemble ubiquitin polymers [20,21,22]. Given that ubiquitination and deubiquitination are regulated in a plethora of biological mechanisms, extensive studies have addressed their roles in cell cycle progression, DNA damage repair, cancer-related cellular signaling, and oncogenesis [23,24,25,26,27,28,29,30]. The human genome encodes approximately 100 DUBs, which can be divided into seven distinct subfamilies [20]. Focusing on ubiquitin C-terminal hydrolases (UCHs), four subfamilies have been identified in humans: UCHL1, UCHL3, UCHL5, and BAP1 (BRCA1-associated protein-1) [20]. The main function of the UCHs is to recognize and hydrolyze the isopeptide bond at the C-terminal glycine of ubiquitin [31]. As previously mentioned, data linking UCH enzymes to oncogenic processes across various tissues have already been reported, but their specific contributions in endometrial cancer are only beginning to be unraveled [28,32,33,34]. Mechanistically, UCHL1 is suggested to promote tumor cell proliferation by stabilizing cell-cycle regulators. A published report focusing on uterine serous cancer found that in these models, UCHL1 binds to and deubiquitinates cyclin B1, thereby preventing its degradation and prolonging cyclin B1 protein half-life [34]. UCHL1-driven accumulation of cyclin B1 mechanistically facilitates unchecked G2/M cell cycle progression and tumor expansion [34,35]. Consistent with this mechanism, shRNA-mediated UCHL1 ablation in serous EC models was shown to deplete cyclin B1, leading to cell cycle arrest and reduced proliferation in in vitro and xenograft models [34]. Increasing evidence suggests that in endometrial carcinoma, the net impact of UCHL1 upregulation is a more aggressive, proliferation-prone tumor phenotype [26,34]. The specific role of UCHL3 (a known homolog of UCHL1) in EC is less well characterized. High *UCHL3* expression was reported in ovarian carcinoma and was associated with poorer clinical outcomes [27]. In ovarian cancer cells, elevated UCHL3 promoted malignancy by deubiquitinating and stabilizing TNF receptor-associated factor 2 (TRAF2), thereby hyperactivating the NF-κB inflammatory pathway [27]. This mechanism was thus reported to enhance cytokine signaling and cancer cell survival [27]. UCH37, also known as UCHL5, functions as a component of the 26S proteasome, where it trims ubiquitin chains from substrates targeted for degradation [20]. This DUB plays an important role in regulating the turnover of signaling proteins and has been implicated in cancer development [20]. In endometrial carcinoma, a recent study identified UCHL5 as an oncogenic factor that drives tumor growth via the Wnt/β-catenin pathway [28]. This study, which analyzed data from the TCGA EC cohort, found UCHL5 overexpression in endometrial tumor tissue compared with adjacent normal endometrium [28]. Furthermore, higher UCHL5 levels were associated with significantly shorter overall survival in patients [28]. The authors also conclude that overexpression of UCHL5 in EC cells accelerates proliferation, promotes cell cycle progression, and inhibits apoptosis, whereas shRNA-mediated knockdown of UCHL5 produces the opposite effect [28]. BRCA1-associated protein 1 (BAP1), a ubiquitin carboxyl-terminal hydrolase, plays a dual role in cancer-related pathological processes [20]. Initially, data suggested that BAP1 was a tumor suppressor as it suppressed the growth of MCF7 breast cancer cells through the BRCA1-related pathway [36]. Furthermore, while germline BAP1 mutations are known to drive a spectrum of malignancies, collectively termed BAP1 cancer syndrome [36], the functional role of this deubiquitinase in endometrial cancer (EC) remains largely unexplored. Crucially, while UPS is a key effector of protein stability, the higher-order coordination of these proteostatic networks during cancer progression remains poorly understood. Emerging evidence suggests that long non-coding RNAs (lncRNAs) act as master regulators of phenotypic switching. This process may be coupled to protein degradation pathways during tumor evolution, yet the global transcriptomic landscape of this relationship has not been comprehensively mapped in endometrial malignancies.

To address this gap, this study utilized unbiased, whole-transcriptome RNA-sequencing (RNA-seq) on a strictly paired cohort of EC patients and patients with atypical endometrial hyperplasia (EIN). Our primary aim was to map the global transcriptomic and proteostatic rewiring that drives the transition from adjacent normal endometrium to localized neoplasia and invasive carcinoma by employing a matched-pair statistical design (tumor vs. adjacent normal). Through this approach, we identify a profound, malignancy-specific dysregulation of the UPS, particularly within the UCHL family of deubiquitinases. We demonstrate that this proteostatic shift is tightly coordinated with specific invasive lncRNA networks, mapping these dual-mechanistic signatures across the distinct molecular and histologic subtypes of endometrial cancer.

## 2. Results

Of the 38 patients, 34 were diagnosed with endometrial cancer, and four were diagnosed with EIN. The mean age at diagnosis was 64 years (range 42–86). Median BMI was 29.5 (range 18.4–42.3). Data regarding final histology, FIGO stage, and molecular subtype are presented in Table 1.

To characterize the comprehensive transcriptional alterations associated with endometrial carcinogenesis, we performed RNA sequencing on a cohort of 76 samples, comprising 38 pairs of tumors and matched adjacent normal endometrial tissues. We assessed the global expression profiles of 17,688 protein-coding mRNAs and 23,362 long non-coding RNAs (lncRNAs).

**Figure 1:**
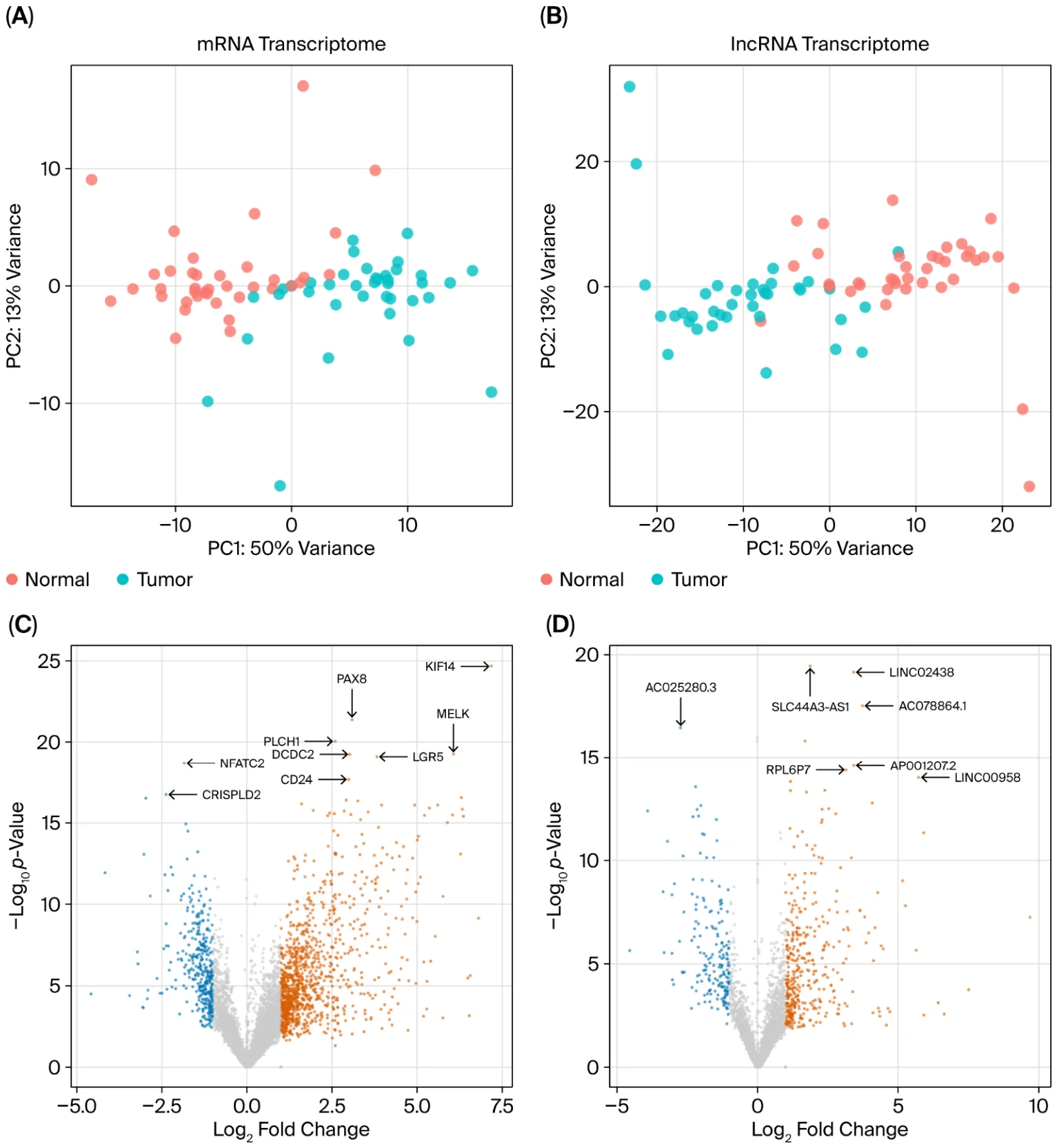
Distinct mRNA and lncRNA transcriptomic profiles in endometrial cancer. (**A**,**B**) Principal component analysis (PCA) of the batch-corrected mRNA (**A**) and lncRNA (**B**) transcriptomes. Scatter plots demonstrate a clear segregation between adjacent normal tissue (pink) and tumor samples (cyan), driven by the underlying neoplastic process. The proportion of variance explained by the first and second principal components (PC1 and PC2) is indicated on the respective axes. (**C**,**D**) Volcano plots illustrating the differential expression of mRNAs (**C**) and lncRNAs (**D**) between tumor and adjacent normal tissues. Orange points represent significantly upregulated transcripts, blue points represent significantly downregulated transcripts, and gray points indicate non-significant alterations (adjusted *p* < 0.05 and |log_2_ fold change| > 1.0). Of the 17,688 total mRNAs analyzed, 1475 were significantly differentially expressed. Similarly, of the 23,362 analyzed lncRNAs, 577 showed significant differential expression. Key top-regulated transcripts are highlighted.

Principal component analysis (PCA) of the batch-corrected transcriptome revealed a distinct separation between tumor and adjacent normal tissue phenotypes, reflecting the underlying neoplastic process (Figure 1A,B). This segregation was evident in the mRNA (Figure 1A) and lncRNA (Figure 1B) compartments, confirming that the regulatory non-coding transcriptome changes to a comparable magnitude to the metabolic and structural shifts observed in the coding transcriptome. While most samples clustered according to disease state, a subset of tumors exhibited transcriptional profiles that partially overlapped with those of adjacent normal tissues, reflecting the inherent molecular heterogeneity and varying degrees of differentiation within the cohort.

Differential expression analysis identified widespread dysregulation of the endometrial transcriptome. Using a stringent significance threshold (Benjamini–Hochberg adj.*p* < 0.05 and ∣log_2_FC∣ > 1), we identified 1475 significantly differentially expressed mRNAs (Figure 1C), representing approximately 8.3% of the quantified coding transcriptome. In parallel, the non-coding landscape showed highly specific modulation, with 577 lncRNAs significantly differentially expressed (Figure 1D). Notably, the fraction of significant transcripts retained after false discovery rate (FDR) correction was remarkably high (1475 adjusted vs. 1482 unadjusted for mRNA; 577 adjusted vs. 578 unadjusted for lncRNA), indicating a robust and consistent transcriptional signal across the cohort despite inter-patient variability.

### 2.1. Divergent Transcriptomic Profiles of UPS Networks Across Molecular Subtypes

We integrated established ubiquitin–proteasome system (UPS) gene sets with empirically derived active ubiquitome signatures to evaluate the transcriptional enrichment of global ubiquitination networks across endometrial cancer molecular subtypes [37]. These custom signatures were extracted from our previously validated tandem ubiquitin binding entity (TUBE) pulldown assays and K48/K63 chain-specific nanobody enrichment profiles [38,39].

**Figure 2:**
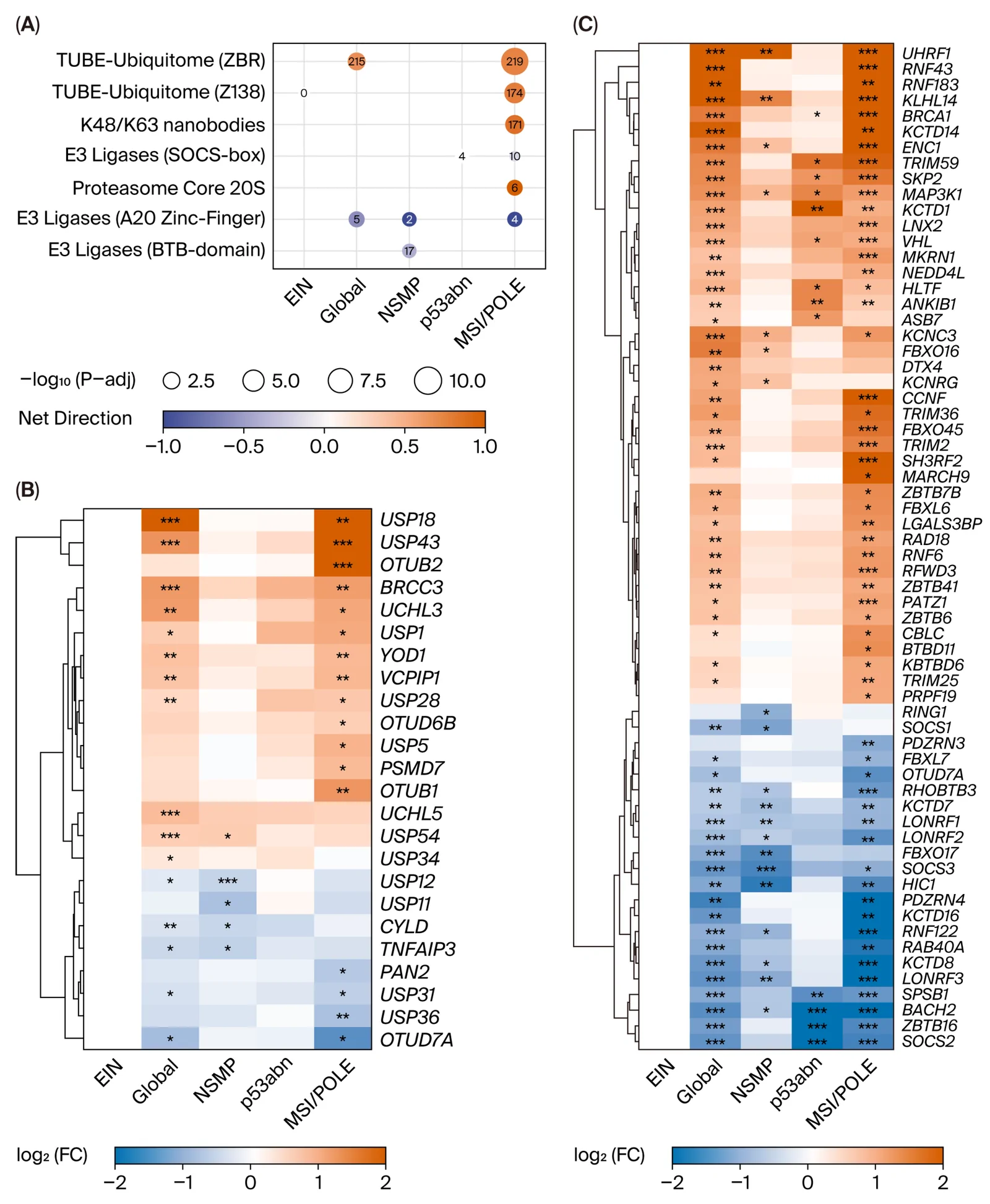
Divergent ubiquitin–proteasome system (UPS) dependencies across endometrial cancer molecular subtypes. (**A**) Functional enrichment of global proteostasis networks and targeted E3 ligase families. Bubble size indicates statistical significance (−log_10_ adjusted *p*-value), and color indicates the net direction of pathway regulation (orange = upregulated; blue = downregulated). Numbers within bubbles denote the total count of significantly differentially expressed genes within that functional category. Hypermutated (MSI/POLE) tumors exhibit a profound upregulation of global polyubiquitination and bulk clearance machinery, as evidenced by the enrichment of empirically derived TUBE-ubiquitome (ZBR/Z138), K48/K63 nanobody signatures, and the 20S Proteasome Core. (**B**,**C**) Hierarchically clustered heatmaps detailing the relative expression (log_2_ fold change) of the top significantly altered deubiquitinases (DUBs) (**B**) and E3 ubiquitin ligases (**C**). The DUB landscape (**B**) highlights a functional division of labor in MSI/POLE tumors, characterized by the upregulation of ubiquitin-recycling enzymes (e.g., UCHL3, UCHL5) to sustain bulk clearance, alongside specific proteases (e.g., USP1) for targeted substrate rescue. The E3 ligase landscape (**C**) reveals concurrent, subtype-specific suppression of critical ligase networks in copy-number high (p53) and NSMP tumors. Asterisks within heatmap cells denote the level of statistical significance for each gene relative to adjacent normal tissue (* adjusted *p* < 0.05, ** adjusted *p* < 0.01, *** adjusted *p* < 0.001).

Strikingly, functional enrichment analysis revealed that hypermutated MSI and POLE tumors exhibit a massive upregulation of broad proteasomal clearance machinery (Figure 2A). This broad transcriptional enrichment of the UPS network was characterized by the profound upregulation of the 20S Proteasome core, alongside the transcriptional enrichment of poly-ubiquitination signatures of poly-ubiquitinated substrates, as evidenced by the intense positive regulation of the transcriptomic projections from custom TUBE (Z138/ZBR) and K48/K63 Poly-Ub nanobody signatures. Conversely, copy-number high (p53abn) and NSMP tumors lacked this global UPS enrichment, underscoring a fundamental divergence in proteostatic states.

To identify the specific enzymatic components underlying these subtype-specific UPS profiles, we interrogated the differential expression of individual deubiquitinases (DUBs) and E3 ubiquitin ligases. Hierarchical clustering of the DUB landscape (Figure 2B) revealed distinct family-specific regulations. In MSI and POLE tumors, members of the UCHL family (specifically UCHL3), which primarily process ubiquitin precursors, were significantly upregulated, mirroring the global proteasomal expansion. Expression of UCHL1 notably remained entirely unaltered across all molecular subtypes. Beyond the UCHL family, the hypermutated MSI and POLE subtypes exhibited profound upregulation of specific ubiquitin-specific proteases (USPs) and OTU-domain DUBs, with USP18, USP43, and OTUB2 emerging as the most significantly enriched targets. Furthermore, the established DNA-damage regulator USP143 was markedly upregulated specifically within the genomically unstable MSI/POLE and Global groups but absent in p53abn and NSMP tumors.

Analysis of the E3 ubiquitin ligase landscape (Figure 2C) further reinforced this subtype divergence. The MSI and POLE tumors demonstrated overall broad upregulation across multiple ligase families, while the NSMP subtypes exhibited significant targeted downregulation of specific ligase networks. Most notably, members of the BTB-domain and A20 Zinc-Finger E3 ligase families were significantly suppressed in the NSMP subtype (Figure 2A).

### 2.2. Subtype-Specific Transcriptional Architectures of Cancer Hallmarks

**Figure 3:**
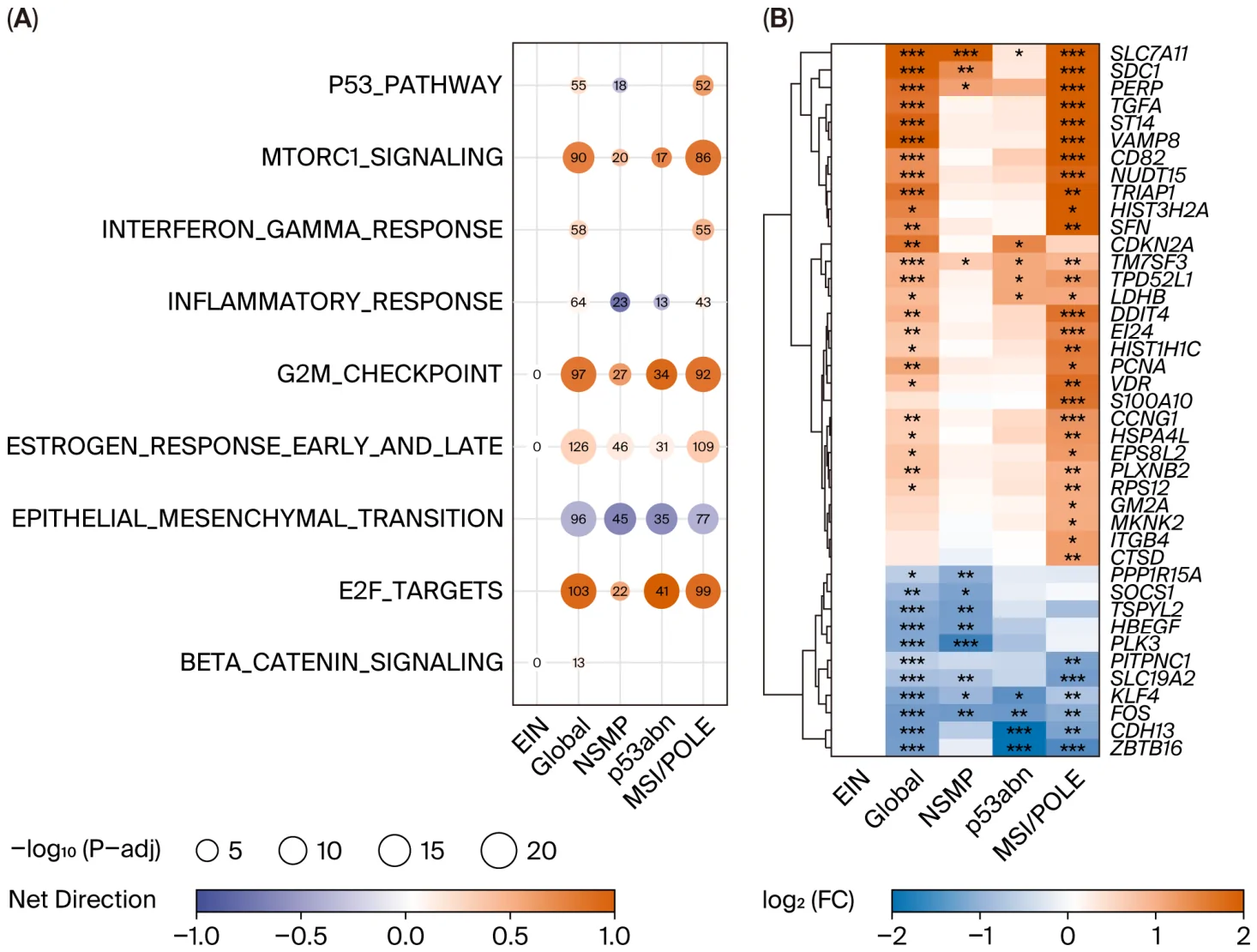
Subtype-specific transcriptional architectures of canonical cancer hallmarks. (**A**) Global functional enrichment of hallmark gene sets across endometrial cancer molecular subtypes. Bubble size indicates statistical significance (−log_10_ adjusted *p*-value), and color indicates the net direction of pathway regulation (orange = upregulated; blue = downregulated). Numbers within bubbles denote the total count of significantly differentially expressed genes within that functional category. Hypermutated (MSI/POLE) and copy-number high (p53abn) tumors exhibit intense yet distinctive transcriptional enrichment signatures across major proliferative, immune, and stress-response networks. Notably, while both exhibit distinct stress responses, coordinated enrichment of the P53 pathway is unique to the MSI/POLE cohort. (**B**) Hierarchically clustered heatmap detailing the relative expression (log_2_ fold change) of the specific, significantly altered genes comprising the P53 pathway. The granular transcriptomic landscape reveals that while the MSI/POLE subtype exhibits strong net activation of the coordinated pathway, the p53abn subtype is characterized by the disjointed dysregulation of individual downstream targets without global pathway enrichment. This reflects the underlying divergence between wild-type DNA-damage responses (MSI/POLE) and mutated p53 signaling (p53abn). Asterisks within heatmap cells denote the level of statistical significance for each gene relative to adjacent normal tissue (* adjusted *p* < 0.05, ** adjusted *p* < 0.01, *** adjusted *p* < 0.001).

Building upon the divergent proteostasis dependencies established in functional networks (Figure 2), we next interrogated the broader transcriptomic signatures across canonical cancer hallmark gene sets. We performed functional enrichment analysis against the MSigDB cancer hallmarks collection to translate transcriptional alterations into biological mechanisms. Consistent with the gene-level analysis, our small subset of pre-cancerous EIN lesions (n = 4) did not exhibit significant enrichment of hallmark pathways compared with adjacent normal tissue (Figure 3A). While the low statistical power of this subset limits definitive conclusions, this preliminary observation generates the hypothesis that major transcriptomic shifts underlying cell cycle entry and metabolic reprogramming primarily consolidate during the transition to invasion rather than as early initiating events.

The landscape in the global tumor cohort was dominated by a massive induction of cell cycle progression signatures, including *E2F Targets* (103 genes) and *G2M Checkpoint* (97 genes), alongside metabolic upregulation via mechanistic target of rapamycin complex 1 (mTORC1) Signaling (90 genes). However, stratification by molecular subtype revealed divergent functional drivers obscured in the global view. The hypermutated MSI/POLE subtype exhibited the most active functional landscape, uniquely characterized by robust immune activation. This included specific enrichment of the Interferon Gamma Response (55 genes) and Inflammatory Response (43 genes), consistent with the high neo-antigen load and lymphocytic infiltration known to define mismatch repair-deficient tumors (Figure 3A).

Crucially, the regulation of the P53 pathway provided functional validation of our molecular classification. The MSI/POLE subtype displayed strong net activation of P53 signaling (52 genes), reflecting the robust physiological response of WT p53 to replication stress (Figure 3A). In stark contrast, the clinically aggressive p53abn subtype failed to show significant enrichment of the coordinated P53 pathway. As revealed by our subsequent granular analysis (Figure 3B), while specific downstream targets within the network are highly dysregulated in p53abn tumors, the pathway as a coordinated whole is not significantly enriched. This absence of a global signal functionally confirms the loss of WT p53 transcriptional activity in this group. Furthermore, while canonical Estrogen Response signatures were robustly upregulated across all invasive subtypes, this classical hormonal dependency was most pronounced in the highly mutated MSI/POLE cohort. Strikingly, Epithelial–Mesenchymal Transition (EMT) pathways were universally suppressed across all tumor subtypes, with the most severe downregulation again observed in the MSI/POLE group. However, rather than definitively indicating a non-classical invasion mechanism, this global reduction in mesenchymal markers is highly likely to reflect the shifting cellular composition of the tissue—specifically, the high epithelial cellularity of the malignant biopsies compared with the naturally stroma-rich adjacent normal endometrium.

We performed a granular interrogation of the P53 pathway (Figure 3B) to delineate the specific gene-level architectures underlying these global hallmark signatures. While the functional enrichment summary indicated strong net positive regulation of the P53 network in the MSI/POLE cohort, hierarchical clustering of the individual genes revealed highly divergent underlying transcriptional networks. The transcriptomic landscape demonstrates that the p53abn subtype, characterized by underlying TP53 driver mutations, exhibits a distinct cluster of specifically dysregulated downstream targets compared with the wild-type p53 MSI/POLE group. For instance, target genes such as CDH13 and ZBTB16 were profoundly suppressed in the p53abn and MSI/POLE subtypes but remained largely unaltered in the NSMP group.

This granular analysis also revealed universally altered targets with subtype-specific magnitudes. Notably, SLC7A11 was significantly upregulated across all endometrial cancer subtypes; however, the magnitude of this induction varied widely. SLC7A11 exhibited the most pronounced upregulation in the NSMP and hypermutated MSI and POLE tumors, whereas it showed a distinctly blunted induction in the p53abn cohort.

### 2.3. Divergent lncRNA Programs Characterize the Contrast Between Normal and Malignant Phenotypes

**Figure 4:**
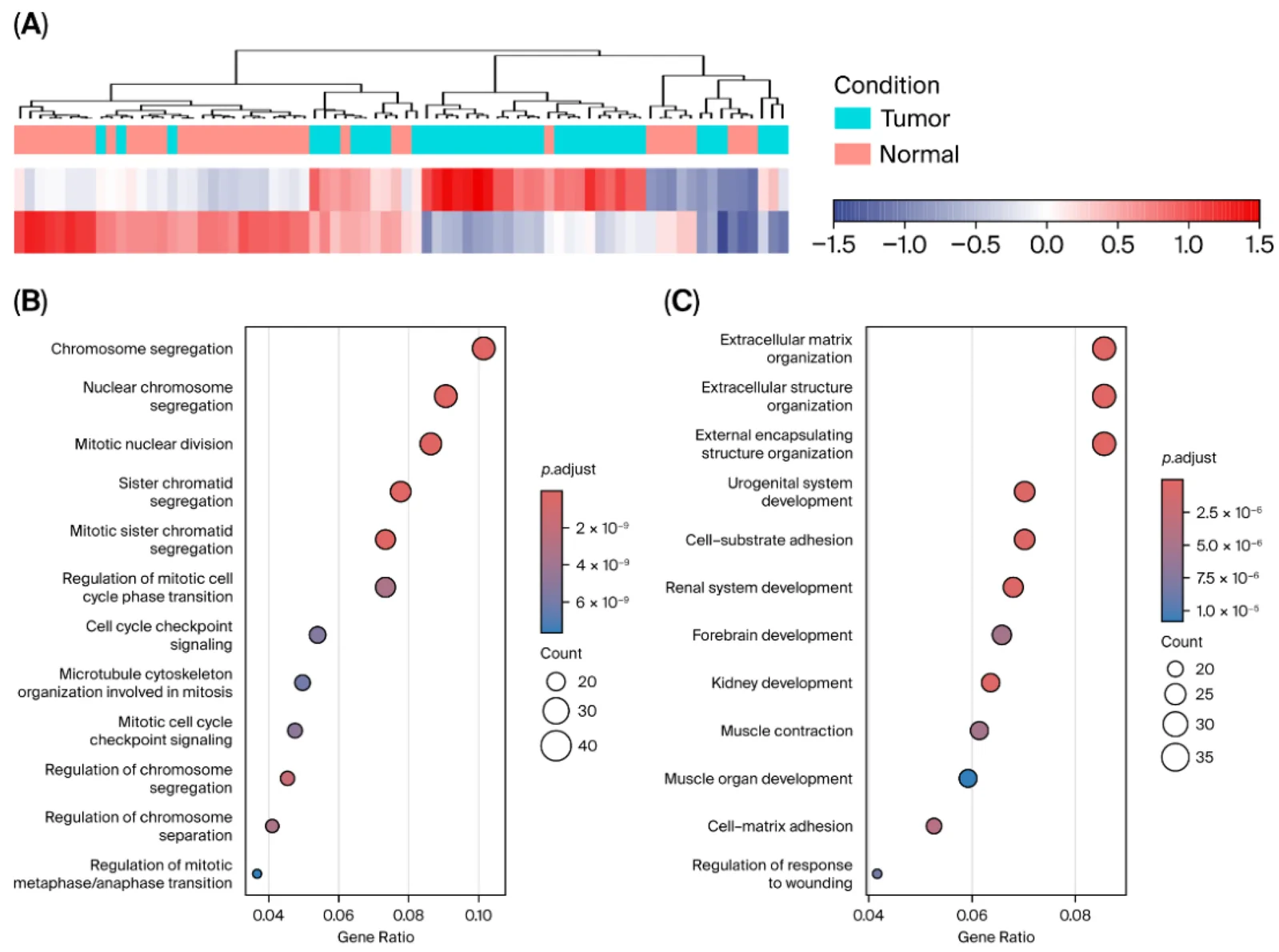

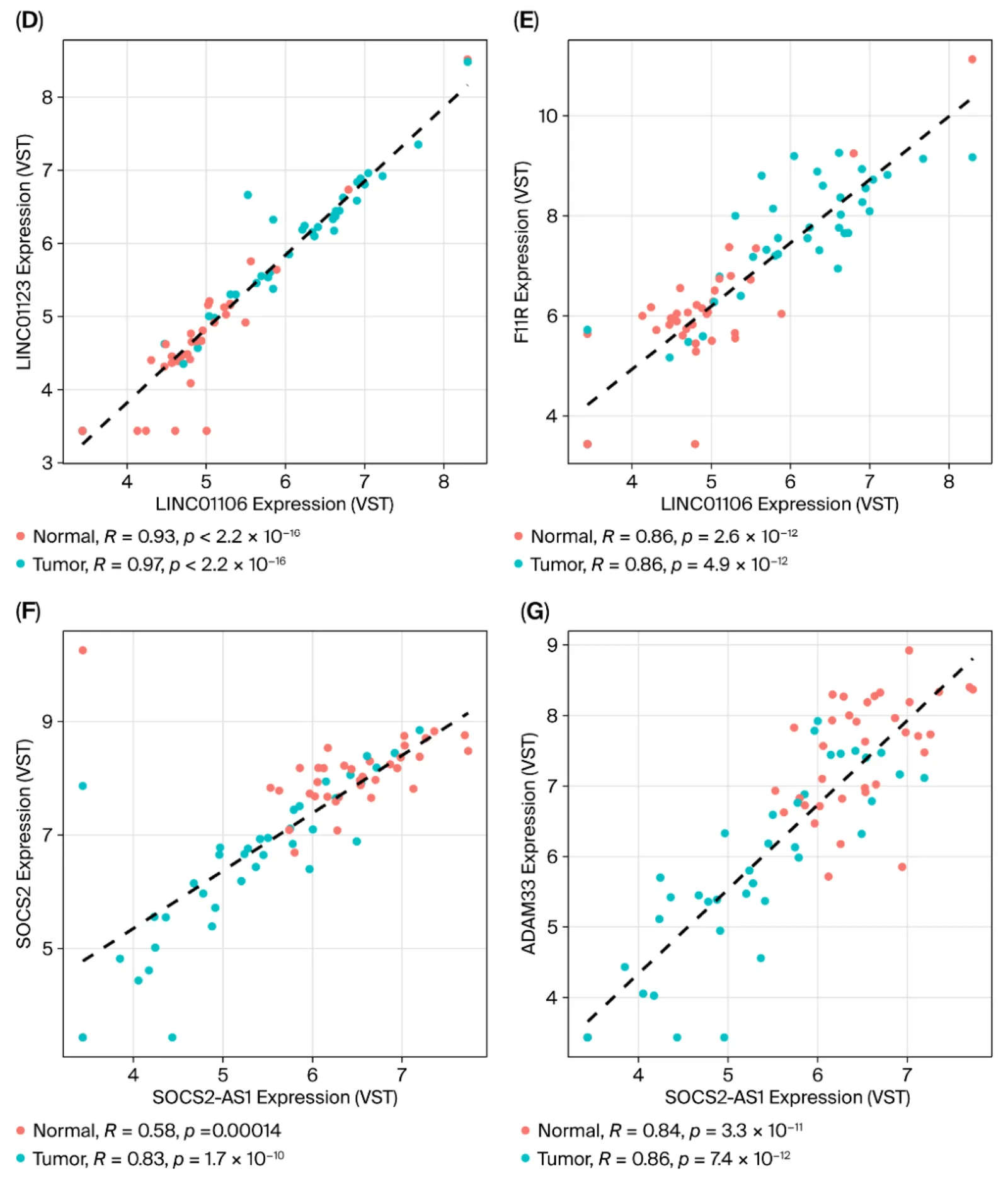
Distinct lncRNA transcriptional modules are associated with proliferative and invasive phenotypes. (**A**) Unsupervised hierarchical clustering (k = 2) of differentially expressed lncRNAs identifies two divergent regulatory programs: a Tumor-Enriched module (Cluster 1) and a Tumor-Suppressed module (Cluster 2). Columns represent individual tissue samples; rows represent transcripts. (**B**,**C**) Functional enrichment analysis (GO) of the two modules. Cluster 1 (**B**) is dominated by mitotic and cell cycle checkpoint pathways, whereas Cluster 2 (**C**) is robustly enriched for extracellular matrix (ECM) organization and adhesion. (**D**,**E**) Co-expression dynamics of the Cluster 1 proliferative module. Scatter plots show near-perfect synchronization between genomic neighbors *LINC01106* and *LINC01123* (**D**) and a strong correlation between *LINC01106* and the mitotic effector *F11R* (**E**), linking this locus to the cell division machinery. (**F**,**G**) Characterization of the *SOCS2-AS1* invasion-associated module (Cluster 2). The lncRNA exhibits stronger trans-coupling with the metalloproteinase *ADAM33* (**G**) than with its local host gene *SOCS2* (**F**), suggesting a functional role in ECM remodeling independent of the canonical cis-regulatory axis. Pearson’s correlation coefficients (R) and *p*-values were calculated independently for the normal (red) and tumor (teal) cohorts.

To define the non-coding landscape of endometrial transformation, we performed global expression profiling, revealing a bifurcation of the lncRNA transcriptome into two distinct regulatory programs (Figure 4A), which reflects upregulated versus downregulated lncRNAs in tumor tissues compared with adjacent normal tissues. The proliferative module (Cluster 1) consisted of transcripts specifically upregulated in the tumor cohort. Functional enrichment mapped this group to Nuclear Division and Chromosome Segregation (Figure 4B). Within this module, we identified a tightly coupled regulatory hub involving *LINC01106* and its genomic neighbor *LINC01123*. These transcripts displayed near-perfect co-expression (R = 0.93/0.97; Figure 4D) and were strongly correlated with the mitotic effector *F11R* (R = 0.86/0.86; Figure 4E), as well as other mRNAs for proteins involved in cell division, suggesting they form a highly co-expressed transcriptional locus associated with the proliferative expansion of the malignant clone. The invasive module (Cluster 2) was characterized by transcripts enriched for extracellular matrix (ECM) organization and cell–matrix adhesion (Figure 4C), reflecting the structural remodeling required for invasion. A central component of this network was *SOCS2-AS1*.

### 2.4. SOCS2-AS1 Emerges as a Key Co-Expressed Node in an Invasion-Associated Network

We further dissected the co-expression dynamics of *SOCS2-AS1* within the invasive module. While the lncRNA showed significant co-expression with its local host gene *SOCS2* (R = 0.58/0.83; Figure 4F), this *cis*-association was notably weaker than its coupling to distal targets. *SOCS2-AS1* exhibited fairly precise synchronization with the metalloproteinase ADAM33 (R = 0.84/0.86, *p* < 10^−11^; Figure 4G), a key mediator of stromal degradation. This preferential *trans*-correlation indicates that the transcriptional profile of SOCS2-AS1 highly synchronized with the broader pro-invasive ECM program, though functional validation is required to distinguish true co-regulation from shared cellular origins within the bulk tumor microenvironment.

## 3. Discussion

This RNA analysis provides further insight into the role of the UCH family in the oncogenic processes driving endometrial cancer. We assessed the global expression profiles of 17,688 protein-coding mRNAs and 23,362 long non-coding RNAs (lncRNAs) in a cohort of 38 patients with endometrial intraepithelial neoplasia (EIN) (n = 4) and endometrial cancer (n = 34). Principal component analysis (PCA) of the batch-corrected transcriptome revealed a distinct separation between tumor and adjacent normal tissue phenotypes, reflecting the underlying neoplastic process (Figure 1A,B). This segregation was evident in the mRNA and lncRNA compartments, confirming that the regulatory non-coding transcriptome changes to a comparable magnitude to the metabolic and structural shifts observed in the coding transcriptome. While most samples clustered according to disease state, a subset of tumors exhibited transcriptional profiles that partially overlapped with those of adjacent normal tissues, reflecting the inherent molecular heterogeneity and varying degrees of differentiation within the cohort. Differential expression analysis identified widespread dysregulation of the endometrial transcriptome. We identified 1475 significantly differentially expressed mRNAs (Figure 1C) using a stringent significance threshold (adj.*p* < 0.05 and log_2_FC > 1), representing approximately 8.3% of the quantified coding transcriptome. In parallel, the non-coding landscape showed highly specific modulation, with 577 lncRNAs significantly differentially expressed (Figure 1D). Notably, the fraction of significant transcripts retained after false discovery rate (FDR) correction was remarkably high, indicating a robust and consistent transcriptional signal across the cohort.

Functional interrogation of these global signatures revealed a classic oncogenic reprogramming event. The upregulated coding transcriptome was dominated by genes involved in high-fidelity cell cycle progression (e.g., CDK1, TOP2A, MKI67, and PCNA), consistent with the hyper-proliferative state of malignant endometrium [19]. Conversely, the downregulated fraction was characterized by the reduced expression of canonical drivers of the epithelial–mesenchymal transition (EMT) and extracellular matrix organization, including TGFB1, COL3A1, ACTA2, and THBS1. Rather than reflecting an active dismantling of the mesenchymal program by the tumor cells, this broad repression in our bulk RNA-seq data likely stems from fundamental differences in tissue architecture, namely, the displacement of normal stroma and fibroblasts by the dense epithelial expansion of the tumor [40]. Epithelial–mesenchymal transition is commonly associated with tumor invasion and metastasis; however, mesenchymal–epithelial transition and plasticity in this process also play a role in several cancers [27,41,42].

Intriguingly, this global shift in the coding transcriptome was mirrored by specific alterations in the UPS, a critical regulator of protein homeostasis, prompting a focused examination of deubiquitinating enzymes (DUBs) as potential drivers of this proteostatic reset. Therefore, we further analyzed transcriptional expression of the UCHL family and its role in the mechanistic processes underlying endometrial cancer. We found that UCHL5 was significantly upregulated in cancer tissues compared with adjacent normal controls, consistent with its known role in stabilizing oncogenic signaling intermediates, including components of the Wnt pathway. Our results are consistent with the already published literature in endometrial cancer [30]. Similarly, our data showed that UCHL3 exhibited robust global upregulation, suggesting that these DUBs are potential drivers of the invasive phenotype.

An exploratory aspect of our research is the preliminary analysis of a small subset of patients with EIN. In contrast to invasive tumors, this specific EIN subset showed no significant dysregulation of *UCHL1*, *UCHL3*, or *UCHL5* compared with adjacent normal tissue. While the sample size precludes definitive claims, we hypothesize that UCHL activation does not act as an early initiating event in endometrial cancer but rather represents a dependency acquired during the transition to invasion, a hypothesis that warrants testing in larger EIN cohorts. High tumor mutational burden is known to generate abundant neoantigens and induce significant cellular stress. Consequently, we hypothesize that MSI and POLE-ultramutated tumors specifically upregulate UCHL3 transcription as a potential mechanism to manage the resulting predicted cellular stress and maintain protein homeostasis. This hypothetical UCHL3 association may also directly contribute to the highly immunogenic microenvironment characteristic of hypermutated tumors. In closely related gynecological malignancies such as ovarian cancer, elevated UCHL3 has been shown to promote malignancy by deubiquitinating and stabilizing TNF receptor-associated factor 2 (TRAF2), thereby hyperactivating the NF-κB inflammatory pathway [43]. This established mechanism elegantly aligns with our transcriptomic profiling (Figure 3A), which revealed unique, massive enrichment of Inflammatory and Interferon Gamma responses specifically within the UCHL3-high MSI/POLE cohort. Conversely, while UCHL5 was significantly upregulated in the global pan-cohort analysis, its upregulation across the individual molecular subtypes did not reach statistical significance after multiple testing correction.

This suggests that the upregulation of UCHL5 is likely a broad transcriptomic feature rather than a subtype-specific marker. Interestingly, UCHL1 levels were not upregulated in any molecular subtype in our cohort, representing a unique profile for endometrial cancer compared with other malignancies [22]. We attribute this discrepancy to the composition of our cohort, which predominantly encompasses early-stage cases. This contrasts with previous reports associating *UCHL1* upregulation specifically with advanced disease and a poor prognosis [28,44].

The preliminary observation that UCHL dysregulation was not detected in our pre-cancerous EIN subset yet was ubiquitous in invasive carcinoma provides an initial framework for the broader molecular history of endometrial cancer. This suggests that the upregulation of proteostatic machinery may serve less as an early initiating driver but rather a necessary adaptation acquired during the explosive growth phase of invasion. To confirm that this all-or-nothing pattern was not limited to the ubiquitin system, we expanded our interrogation to the global functional landscape.

### 3.1. Functional Evolution from Quiescence to Aggressive Proliferation

Our transcriptomic characterization provides a descriptive roadmap of the divergent transcriptomic states between precursor lesions and invasive carcinoma. A notable preliminary observation in our cohort is the lack of significant hallmark enrichment in the EIN subset (Figure 3). Despite being histologically distinct from normal endometrium, the absence of enriched classic oncogenic hallmarks in these samples offers a preliminary hypothesis regarding the trajectory of transcriptomic accumulation. This suggests the possibility that the acquisition of the malignant phenotype, characterized by the massive induction of E2F and G2M checkpoints, may represent a distinct reprogramming event that consolidates near the invasion boundary, though larger studies are required to rule out gradual molecular accumulation.

### 3.2. Genomic Instability Strongly Defines the Immune and Proteostatic Landscape

Stratification by molecular subtype revealed that genomic instability is the primary determinant of the tumor microenvironment. The MSI and POLE-ultramutated subtypes were uniquely defined by robust Interferon Gamma and Inflammatory Response signatures. This functional readout confirms that the high neo-antigen load characteristic of these tumors highly correlates with signatures of active immune surveillance.

Crucially, this immune-active environment likely imposes significant cellular stress, providing a mechanistic context for our observation of UCHL3 and general overall UPS upregulation in these specific subtypes. We hypothesize that hypermutated tumors exhibit a strong transcriptomic association with UCHL3 to manage the cellular stress associated with high mutational burdens and active cytokine signaling. This identifies the transcriptomic enrichment of the proteasome–ubiquitin system as a candidate target requiring further functional validation in these immunologically hot subtypes.

Our functional analysis may help to resolve the divergent impact of TP53 status across molecular subtypes. We observed a striking dichotomy in p53 signaling: MSI and POLE-ultramutated tumors that retain wild-type TP53 exhibited robust p53 pathway activation, reflecting a functional stress response to high mutational burden. In sharp contrast, the copy-number high (p53abn) subtype displayed no significant enrichment of p53 signaling. This functional silence confirms that the defining TP53 mutations in this group result in a loss of canonical transcriptional activity. This inert phenotype also extended beyond p53; compared with the hypermutated subtypes, the p53abn group showed diminished immune activation and weaker enrichment of estrogen response signatures, painting a picture of a tumor driven by genomic instability rather than inflammatory or hormonal signaling [1].

However, our granular analysis of the P53 pathway (Figure 3B) revealed that this lack of global enrichment in p53abn tumors obscures a highly specific, rewired downstream target network. While MSI/POLE and p53abn tumors utilize fundamentally divergent transcriptional architectures to manage their respective genomic stresses, we identified critical nodes of convergent evolution. The well-established tumor suppressors CDH13 (which restricts cellular invasion) and ZBTB16 (which restrains cell cycle progression) specifically were profoundly suppressed in the hypermutated and p53abn cohorts. In contrast, they remained largely unaltered in the less aggressive NSMP group. This shared suppression suggests that despite possessing different initiating drivers, both highly aggressive subtypes undergo convergent transcriptional rewiring to dismantle the same critical tumor-suppressive checkpoints.

Beyond direct cell cycle regulation, this granular transcriptomic analysis revealed a ubiquitous upregulation of SLC7A11 across all invasive subtypes, highlighting a universal dependency on glutathione-mediated antioxidant defense against ferroptosis. Interestingly, the magnitude of SLC7A11 induction was highly subtype-dependent. The extreme SLC7A11 upregulation observed in hypermutated MSI/POLE tumors elegantly aligns with their massive global proteasomal expansion (Figure 2A). In established models, the cellular stress associated with high neoantigen loads has been shown to activate the Unfolded Protein Response (UPR), a pathway that can upregulate *SLC7A11* transcription via ATF4 [43]. Hyper-upregulation of SLC7A11 also provides a critical resistance mechanism against interferon-γ-induced ferroptosis [45], a key threat in their highly immunogenic microenvironment. Conversely, while p53abn tumors also upregulated SLC7A11, this induction was notably lower than in hypermutated tumors. This moderate upregulation likely results solely from the loss of wild-type p53, which canonically represses SLC7A11 transcription [43]. Because p53abn tumors lack the massive proteotoxic and inflammatory stress that drives extreme *SLC7A11* expression in the MSI/POLE group, their antioxidant response remains comparatively blunted.

Invasion beyond classical EMT is perhaps the most intriguing observation from our hallmark analysis. However, interpreting this as “invasion beyond classical EMT” must be performed with extreme caution. In bulk transcriptomics, this reduced mesenchymal signature is heavily influenced by the lower stromal/fibroblast abundance in highly cellular tumor biopsies relative to adjacent normal endometrium. Future single-cell sequencing or computational cell-type deconvolution will be strictly required to determine whether true alternative dissemination mechanisms are at play at the cellular level or whether this signature purely reflects epithelial dominance in the sample compartment. This prompted us to investigate the non-coding transcriptome and hypothesize that long non-coding RNAs (lncRNAs) function as the distinct regulatory scaffolds that orchestrate these specialized invasive programs.

This hypothesis is strongly supported by our observation of subtype-specific rewiring within the E3 ubiquitin ligase landscape (Figure 2C). While hypermutated tumors broadly upregulated ligase families, the NSMP subtype exhibited a unique, targeted suppression of the BTB-domain and A20 Zinc-Finger families, providing a distinct molecular signature for this traditionally difficult-to-characterize cohort. Furthermore, across the broader ligase landscape, we identified a striking paralog-specific suppression of the Suppressor of Cytokine Signaling (SOCS) ligase adaptors. SOCS1 and SOCS3 were severely downregulated in NSMP tumors, whereas SOCS2 was specifically suppressed in the hypermutated MSI/POLE and p53abn groups. Because SOCS2 acts as a critical negative regulator of cytokine signaling [46], its targeted loss is likely to synergize with the hyperactive inflammatory state we observed in these subtypes. The discovery of this targeted E3 ligase suppression crucially provided the ideal mechanistic context to interrogate the non-coding transcriptome.

### 3.3. Divergent lncRNA Programs Are Tightly Coupled to the Switch from Homeostasis to Invasion

To define this non-coding landscape, we performed global expression profiling, revealing a striking bifurcation of the lncRNA transcriptome into two distinct regulatory programs (Figure 4A). This clustering segregated the transcriptome into those silenced during transformation and those specifically activated in the tumor.

The proliferative module (Cluster 1) consisted of transcripts specifically upregulated in the tumor cohort. Functional enrichment mapped this group to Nuclear Division and Chromosome Segregation (Figure 4B), aligning with the high-proliferative state observed in our global coding analysis. Within this module, we identified a tightly coupled regulatory hub involving LINC01106 and its genomic neighbor LINC01123. These transcripts displayed near-perfect co-expression (Normal R = 0.93/Tumor R = 0.97; Figure 4D) and were strongly correlated with the mitotic effector F11R (Normal R = =0.86/Tumor R = 0.86; Figure 4E), as well as other mRNAs encoding cell division machinery. This suggests that LINC01106 and LINC01123 may represent a highly co-expressed transcriptomic locus associated with the proliferative expansion of the malignant clone.

In contrast, the invasive module (Cluster 2), which contained transcripts downregulated in tumors relative to adjacent normal tissue, was paradoxically enriched for Extracellular Matrix (ECM) Organization and Cell–Matrix Adhesion (Figure 4C). This confirms that the loss of epithelial architecture, which we observed globally, is not a passive degradation but a regulated program. A central component of this network was SOCS2-AS1. SOCS2-AS1 defines a highly correlated invasion-associated network. To validate the co-expression dynamics of this invasive module, we analyzed the functional connectivity of SOCS2-AS1. While the lncRNA showed significant co-expression with its local host gene SOCS2 (Normal R = 0.58/Tumor R = 0.83; Figure 4F), this *cis*-association was notably weaker than its coupling to distal targets involved in matrix remodeling. SOCS2-AS1 exhibited precise synchronization with the metalloproteinase ADAM33 (Normal R = 0.84/Tumor R = 0.86, *p* < 10^−11^; Figure 4G), a key mediator of stromal degradation. This preferential *trans*-correlation suggests that while SOCS2-AS1 maintains a regulatory link with the SOCS2 locus, its functional scope extends beyond local *cis*-regulation, acting as a key transcript strongly associated with the broader pro-invasive ECM program.

Regarding limitations, we acknowledge that the relatively small sample size may limit the generalizability of our findings. Despite this, all pathological analysis of the specimens was performed by one expert gynecologic pathologist and reviewed by another, thus ensuring a highly accurate diagnosis and reproducibility. We also acknowledge that given the small number of EIN patients (n = 4), the findings in this subgroup may need to be interpreted with caution. While this matched-pair approach stringently controls for inter-patient genetic and baseline variability, recent pan-cancer transcriptomic analyses have definitively established that histologically normal adjacent to tumor (NAT) tissue represents a unique intermediate state rather than a purely healthy baseline [47]. Adjacent tissues are frequently subject to field cancerization effects, harboring early molecular reprogramming, or are influenced by pro-inflammatory paracrine signaling from the local tumor microenvironment. Consequently, our transcriptomic characterization of the “normal” baseline must be interpreted specifically within this tumor-adjacent context. Future investigations should aim to validate our key molecular findings in an independent cohort of non-oncological patients to further corroborate the robustness of these tumor-specific shifts. Additionally, because our network associations are derived from bulk tissue transcriptomics, strong lncRNA-mRNA correlations may partly reflect shifts in tumor microenvironment cellular composition (e.g., varying proportions of stromal and epithelial cells) rather than intracellular co-regulation. Future single-cell RNA-sequencing and targeted in vitro functional assays (such as knockdowns or RNA-FISH) are essential to definitively establishing direct regulatory relationships. Finally, a major limitation of this study is that our conclusions regarding the ubiquitin–proteasome system and specific UCHL family members are derived exclusively from transcriptomic data. While we utilized empirically derived TUBE and nanobody gene sets, these represent indirect transcriptomic projections rather than direct measurements of ubiquitination or protein accumulation. Our assertions regarding the activity of these UPS components and enzymatic dependencies remain strictly hypothetical based on transcriptional predictions because we did not perform protein-level functional validation (such as Western blotting or immunohistochemistry) for key targets such as UCHL3 or UCHL5. Future functional studies are required to confirm whether these transcriptomic shifts successfully translate into functional therapeutic targets in hypermutated endometrial tumors.

In conclusion, our study adds further data to help redefine the molecular landscape of endometrial carcinoma as comprising distinct transcriptomic signatures associated with two opposing functional states: a proliferative growth program driven by mitotic checkpoints and an invasive program, strongly associated with distinct lncRNA networks, such as *SOCS2-AS1*.

Crucially, the sustainability of these aggressive phenotypes appears to rely on the precise regulation of protein homeostasis. We identify a profound rewiring of the broader UPS, encompassing diverse E3 ligases and deubiquitinating enzymes, as the essential enzymatic machinery supporting this high-turnover state. Within this network, the global upregulation of *UCHL5* across the cohort, alongside the targeted transcriptional upregulation of *UCHL3* specifically in hypermutated tumors, emerges as a prominent transcriptomic feature. This suggests a model in which lncRNA-associated transcriptional reprogramming guides the tumor’s phenotypic trajectory. At the same time, concurrent UPS dysregulation provides the necessary proteostatic capacity to manage the resulting proteotoxic stress. Consequently, targeting these localized UPS vulnerabilities, particularly the UCHL axis, may offer a potent therapeutic avenue to dismantle the protein stability required for the proliferative expansion and invasive dissemination of endometrial cancer.

## 4. Material and Methods

### 4.1. Data Collection

Formalin-fixed paraffin-embedded tissue blocks of 38 consecutive patients diagnosed between January 2022 and February 2024 with endometrial cancer (n = 34) and endometrial intraepithelial neoplasia (EIN, n = 4) at a tertiary academic hospital were retrieved. Informed consent was obtained from all patients. Biopsy samples were prioritized due to a higher potential for tumor cells (from hysteroscopy, curettage, or Pipelle biopsy). If these tissue samples were not available, tissue from the surgical specimen containing the primary tumor was used. The adjacent normal endometrium of every patient was also obtained.

### 4.2. DNA Sequencing

POLE somatic mutations were detected at an external, certified laboratory using Sanger sequencing. Mutation screening of exons 9, 11, 13, and 14 of the *POLE* gene (12q24.33, OMIM#174762, NM_006231.3, LRG_789t1) was conducted in samples with tumor content higher than 20% and subsequently analyzed with Sanger sequencing. Exonic regions of interest were amplified via polymerase chain reaction (PCR) with flanking intronic primers and sequenced using an automatic sequencer (ABI Prism 3100-Avant Capillary Array, 36cm, Applied Biosystems, Waltham, MA, USA). Data was evaluated using the DNA Sequencing Analysis Software 6™ Version 6.0 (Applied Biosystems).

### 4.3. Immunohistochemistry

MMRd status was determined with immunohistochemistry (IHC), evaluating the presence or absence of four proteins involved in the mismatch repair pathway: MLH1, PMS2, MSH2, and MSH6. p53 abnormalities were also analyzed using IHC. A representative slide of each sample was selected, and then three-micron-thick serial paraffin sections were processed via IHC using an automated immunostainer (Ventana BenchMark Ultra, Shantou, China). Each IHC marker was examined with light microscopy by two expert pathologists. Regarding MMR enzyme immunohistochemistry, cases were scored as “retained” if uniform intact nuclear staining for the protein was observed. In contrast, MMRd tumors showed no intact nuclear staining. Abnormal p53 staining was recognized either by overexpression (diffuse strong staining of >75% tumor cell nuclei) or the complete absence of staining of tumor cell nuclei in the presence of an intact internal control. p53 wild-type tumors were characterized by scattered nuclear staining with a mixture of negative, weak, and strong staining of tumor cell nuclei.

### 4.4. RNA Extraction, Library Preparation, and Sequencing

Total RNA was extracted from FFPE tissue sections using the RNAprep Pure FFPE Kit (Tiangen, Beijing, China). Prior to extraction, samples were deparaffinized using xylene and ethanol washes. The tissue was subjected to Proteinase K digestion at 56 °C for 1 h for lysis, followed by a 90 °C incubation for 1 h to reverse formalin cross-linking. The RNA concentration was quantified using a Qubit fluorometer, and RNA integrity was evaluated utilizing the DV200 metric via the Agilent 5400 platform. Samples with a DV200 > 30% were advanced to library preparation. An input of up to 500 ng of total RNA was used for library construction. In sequencing library construction, ribosomal RNA was depleted from total RNA, and the resulting RNA was fragmented. Directional libraries were prepared by synthesizing first-strand cDNA using random hexamers, followed by second-strand cDNA synthesis incorporating dUTPs. Following end repair, A-tailing, and adapter ligation, libraries were digested with the USER enzyme, amplified, and purified. High-throughput sequencing was performed on the Illumina NovaSeq X Plus platform (Illumina, San Diego, CA, USA) to generate 150 bp paired-end reads, targeting a minimum depth of 15 Gb of raw data (approximately 100 million raw reads) per sample.

### 4.5. Data Quality Control and Pre-Processing

Raw sequencing reads were subjected to rigorous quality control to ensure data integrity. Low-quality reads and artifacts were filtered using a three-step procedure: (1) reads containing sequencing adapters were removed; (2) reads with an undetermined base (N) content exceeding 0.1% were discarded; and (3) low-quality reads, defined as those where more than 50% of bases possessed a Quality (Phred) score of ≤5, were excluded. The resulting high-quality clean data was used for all downstream analyses.

### 4.6. Read Alignment and Transcript Assembly

Clean reads were mapped to the GRCh38 human reference genome (Ensembl assembly) using HISAT2, a splice-aware aligner optimized for precise junction discovery. Alignment was performed at the genome level to facilitate the detection of alternative splicing events, fusion genes, and novel isoforms. Following alignment, assembled transcripts were reconstructed using StringTie (https://github.com/gpertea/stringtie, accessed on 5 October 2026) by merging mapping information from all samples. This process reconstructed the transcriptome, optimized the start and end positions of known transcripts, and identified novel transcriptional units (lncRNAs and mRNAs) compared with the reference annotation. The final output was generated in GTF format for downstream quantification.

### 4.7. Differential Expression Analysis and Subtype Stratification

Differential gene expression analysis was conducted using the DESeq2 package [44]. A paired design modeling patient identity as a blocking factor (design = ~PatientID + Condition) was implemented for all differential expression comparisons to rigorously control for inter-patient heterogeneity and baseline expression variability across the cohort. To distinguish universal transcriptomic shifts from subtype-specific mechanisms, analyses were performed using a hierarchical strategy: Global transcriptome analysis: To identify pan-endometrial markers, a global paired comparison was performed across the full cohort, comparing each tumor sample with its matched adjacent normal endometrial tissue while accounting for patient identity as a blocking factor. Subtype-specific stratification: The cohort was stratified based on the ProMisE/TCGA molecular classification. The following paired comparisons were performed: (a) copy-number low/NSMP: paired comparison of NSMP tumors vs. matched adjacent normal tissues (n = 16 pairs); (b) copy-number high/p53abn: paired comparison of p53-mutant tumors vs. matched adjacent normal tissues; (c) hypermutated/ultramutated (MSI/POLE): paired comparison of MSI/POLE tumors vs. matched adjacent normal tissues; and (d) pre-cancerous lesions (EIN): paired comparison of endometrial intraepithelial neoplasia (EIN) samples vs. matched adjacent normal tissues to identify early initiating events.

For all comparisons, the Benjamini–Hochberg (BH) procedure was applied to control the false discovery rate (FDR). To generate accurate log_2_ fold change estimates for genes with low counts or high dispersion, log fold change shrinkage was applied using the lfcShrink function [48] with the apeglm estimator. Genes were considered significantly differentially expressed if they met the criteria of shrunken log_2_ fold change >1.0 and adjusted *p*-value < 0.05.

### 4.8. Functional Enrichment Analysis

To identify dysregulated biological pathways, Functional Enrichment Analysis was performed using the hypergeometric function in R4.6.1 [49]. Genes were ranked by the Wald statistic from the DESeq2 analysis. Enrichment was tested against the Molecular Signatures Database (MSigDB) cancer hallmark gene sets. Pathways with an adjusted *p*-value < 0.05 and Normalized Enrichment Score (NES) reflecting consistent directionality were considered significant.

### 4.9. Unsupervised Clustering and lncRNA Module Identification

To characterize global patterns of non-coding transcriptional dysregulation, we analyzed the expression profiles of all long non-coding RNAs (lncRNAs) identified as differentially expressed (∣log_2_FC∣ > 1, *p*adj < 0.05) between tumor and adjacent normal tissues. Variance-stabilized transformation (VST) expression values were extracted, and unsupervised hierarchical clustering was performed using Euclidean distance and Ward’s linkage method (implemented via the pheatmap R package).

To define distinct regulatory modules, the resulting dendrogram was partitioned into k = 2 primary clusters using the cutree function. This segregated the non-coding transcriptome into a “Tumor-Enriched” signature (Cluster 1; upregulated in malignant samples) and a “Tumor-Suppressed” signature (Cluster 2; highly expressed in adjacent normal tissue).

### 4.10. Long Non-Coding RNA (lncRNA) Functional Enrichment Analysis

Biological functions associated with each lncRNA module were interrogated using Gene Ontology (GO) enrichment analysis. For each cluster, the constituent genes were queried against the GO Biological Process database using the clusterProfiler R package. Enrichment significance was assessed using a hypergeometric test, with *p*-values adjusted for multiple comparisons (Benjamini–Hochberg method, *p*adj < 0.05). The top enriched pathways were visualized as dot plots and ranked by gene ratio and statistical significance.

### 4.11. Co-Expression and Trans-Regulatory Network Analysis

To infer the potential regulatory scope of specific lncRNA hubs (e.g., *SOCS2-AS1* and *LINC01123*), we performed a global co-expression analysis across the entire patient cohort (N = 38 pairs of tumors/EIN and matched adjacent normal endometrial tissues). Pearson’s correlation coefficients (R) were calculated between the VST expression profile of the candidate lncRNA and all protein-coding transcripts (mRNAs) in the dataset.

Significant functional partners were defined using high-fidelity correlation thresholds (∣R∣ > 0.8, *p* < 0.0001), yielding 119 correlated transcripts for expression Cluster 1 and 60 for Cluster 2. In the *SOCS2-AS1* invasive module, *trans*-regulatory relationships were prioritized by comparing the correlation strength of distal targets (e.g., *ADAM33*) against the local host gene (*SOCS2*). Linear regression analysis was visualized using ggplot2 and ggpubr, with Pearson’s correlation statistics (R, P) displayed directly on the plots.

### 4.12. Principal Component Analysis

Principal component analysis (PCA) was performed to assess global sample clustering and identify outliers. Raw count data were normalized using the Variance Stabilizing Transformation (VST) within the DESeq2 package. Given the paired experimental design, inter-patient heterogeneity accounted for most of the variance in the unadjusted dataset. To visualize the biological variation attributable to the disease state (tumor vs. adjacent normal) independent of patient baseline characteristics, the patient effect was modeled as a batch factor and removed from the VST-normalized matrix using the remove Batch Effect function from the limma package [50]. The top 500 genes with the highest variance in the batch-corrected data were used to compute the principal components.

### 4.13. Unsupervised Clustering and Visualization

Hierarchical clustering was performed using the pheatmap package. Top variable genes were selected based on variance across the cohort. Clustering distances were calculated using Euclidean distance, and linkage was performed using the complete linkage method.

## Figures and Tables

**Table 1:** Patients’ characteristics.

Characteristic	Value
Age (years)	
Median (range)	64 (42–86)
Body mass index (kg/m^2^)	
Median (range)	29.5 (18.4–42.3)
Histology, n (%)	(N = 38)
Endometrial intraepithelial neoplasia (EIN)	4 (11)
Endometrioid	26 (68)
Non-endometrioid	8 (21)
Molecular profile, n (%)	(N = 34)
POLEult (ultramutated)	3 (9)
MMRd (mismatch repair-deficient)	6 (18)
NSMP (no specific molecular profile)	16 (47)
p53abn (p53 abnormal)	9 (26)
FIGO 2009 Stage, n (%)	(N = 34)
Stage I	28 (82)
Stage II	0 (0)
Stage III	5 (15)
Stage IV	1 (3)

Note: Molecular profiling and FIGO 2009 staging apply exclusively to the invasive endometrial carcinoma cases (N = 34) and exclude pre-cancerous EIN lesions (N = 4).

## Data Availability

The data discussed in this publication have been deposited in NCBI’s Gene Expression Omnibus [45] (and are accessible through GEO Series accession number GSE327792) (https://www.ncbi.nlm.nih.gov/geo/query/acc.cgi?acc=GSE327792, accessed on 1 May 2026). In addition, the original contributions presented in this study are included in the article. Further inquiries can be directed to the corresponding authors.

## Abbreviations

The following abbreviations are used in this manuscript

ATF4	Activating transcription factor 4
BH	Benjamini–Hochberg
DUB	Deubiquitinase
DNA	Deoxyribonucleic acid
EC	Endometrial cancer
ECM	Extracellular matrix
EIN	Endometrial intraepithelial neoplasia
E1/E2/E3	Ubiquitin-activating/-conjugating/-ligase enzymes
EMT	Epithelial–mesenchymal transition
FDR	False discovery rate
FC	Fold change
FIGO	International Federation of Obstetrics and Gynecology
GO	Gene ontology
GTF	Gene transfer format
IHC	Immunohistochemistry
lncRNAs	Long non-coding RNAs
MMR	Mismatch repair
MMRd	Mismatch repair-deficient
MSI	Microsatellite instability
MSigDB	Molecular Signatures Database
mTORC1	Mechanistic target of rapamycin complex 1
NES	Normalized enrichment score
NF-κB	Nuclear factor kappa B
NSMP	No specific molecular profile
OMIM	Online Mendelian Inheritance in Man
PCA	Principal component analysis
PC	Principal component
PCR	Polymerase chain reaction
POLE	DNA polymerase epsilon
ProMisE	Proactive Molecular Risk Classifier for Endometrial Cancer
RT-PCR	Reverse transcription polymerase chain reaction
RNA	Ribonucleic acid
shRNA	Short hairpin RNA
TCGA	The Cancer Genome Atlas
TP53	Tumor protein p53 (gene)
TRAF2	TNF receptor-associated factor 2
TUBE	Tandem ubiquitin binding entity
UCH	Ubiquitin C-terminal hydrolase
UCHL	Ubiquitin C-terminal hydrolase L
UCHL1	Ubiquitin C-terminal hydrolase L1
UCHL3	Ubiquitin C-terminal hydrolase L3
UCHL5	Ubiquitin C-terminal hydrolase L5
UPS	Ubiquitin–proteasome system
UPR	Unfolded protein response
VST	Variance stabilizing transformation
WT	Wild type
